# Granulocyte-colony stimulating factor drives the *in vitro* differentiation of human dendritic cells that induce anergy in naïve T cells

**DOI:** 10.1002/eji.201040659

**Published:** 2010-09-21

**Authors:** Maura Rossetti, Silvia Gregori, Maria Grazia Roncarolo

**Affiliations:** 1 San Raffaele Telethon Institute for Gene Therapy (HSR-TIGET), Department of Regenerative Medicine, Stem Cells and Gene Therapy, San Raffaele Scientific InstituteMilan, Italy; 1 Vita-Salute San Raffaele UniversityMilan, Italy

**Keywords:** DC, G-CSF, IL-10, Tolerance, Treg

## Abstract

G-CSF is a modulator of T-cell and DC functions. Previous reports show that monocytes from G-CSF-treated (post-G) healthy donors differentiate into tolerogenic DC *in vitro* in the presence of autologous serum, containing high levels of IL-10 and IFN-α, and in turn induce type 1 Treg (Tr1) cells. However, the direct effect of G-CSF on DC differentiation was not investigated. Here, we show that monocytes differentiated in the presence of exogenous G-CSF (G-DC) remain CD14^+^CD1a^−^, but acquire a DC-like morphology, express CD83 and CD86 and low levels of the tolerogenic markers Ig-like transcript (ILT)4 and HLA-G. G-DC spontaneously produce IL-10 and, upon stimulation, low levels of IL-12. G-DC display low stimulatory capacity and induce anergy in naïve T cells, but do not confer suppressive function. Therefore, *in vitro* differentiation of monocyte-derived DC in the presence of G-CSF can replicate some but not all features of post-G DC. These findings indicate that the tolerogenic properties of G-CSF do not exclusively reside in its direct effect on DC, which in turn induce T-cell anergy, but also in its ability to generate a tolerogenic milieu *in vivo*, which is necessary for Tr1 cell induction and cannot be replicated *in vitro.*

## Introduction

G-CSF was initially identified as a growth factor for neutrophils 1. Inflammatory stimuli can induce G-CSF production from bone marrow stromal cells, endothelial cells, macrophages and fibroblasts. The G-CSF receptor (G-CSFR, CD114) is expressed on early myeloid progenitors, mature granulocytes, monocytes/macrophages 1, endothelial cells and other non-hematopoietic tissues 2, and also on human T and B cells 3–5. G-CSF induces the proliferation of granulocyte progenitors and activates the effector functions of mature neutrophils 1; moreover, G-CSF induces the proliferation and the differentiation of HSC. Consequently, its current major clinical applications are the acceleration of neutrophil reconstitution after myelo-suppression and the mobilization of bone marrow HSC 6. G-CSF also induces expansion 7–9 and enhancement of phagocytosis 8 of the monocyte/macrophage system.

G-CSF modulates T-cell and DC functions 10,11. PBMC of G-CSF-treated healthy volunteers show a reduction of pro-inflammatory cytokines upon LPS stimulation 7,12–17. The proliferation of T cells from G-CSF-treated patients 9 and healthy stem cell donors 18,19 is profoundly reduced, due to a G1 arrest in T-cell cycle 20,21. This modulation might be indirectly mediated by down-regulation of co-stimulatory molecules and enhancement of IL-10 production by monocytes 18,19,22,23. Moreover, CD4^+^ T cells from G-CSF-mobilized stem cell donors are able to suppress allo-proliferative responses of autologous T cells in a cell contact-independent manner, acquiring a type 1 Treg (Tr1)-like cytokine profile 24. Importantly, direct exposure of human T cells to G-CSF *in vitro* does not induce Tr1 cell differentiation 19,24,25. By contrast, *in vivo* administration of G-CSF in mouse models promotes transplantation tolerance through Tr1 cell induction 26, and has been proved to be protective in several disease models 27–30.

DC are highly specialized APC with unique capacity to activate naïve and memory T cells. In addition, DC are implicated in the maintenance of peripheral tolerance. G-CSF preferentially mobilizes plasmacytoid DC that, in turn, skew T-cell differentiation toward a Th2 phenotype 31. Moreover, CD14^+^ monocytes, in the presence of autologous serum from G-CSF-mobilized healthy donors (post-G serum), containing high levels of IL-10 and IFN-α, give rise to tolerogenic Tr1-inducing HLA-DR^+^CD86^+^CD80^+^CD83^+^IL-12^low^ DC (post-G DC) 32. Similarly, tolerogenic APC precursors able to induce IL-10-producing Treg arise in mice after G-CSF treatment 33. Altogether, these findings indicate that G-CSF *in vivo* is an inducer of IL-10, which is implicated in the differentiation and function of tolerogenic DC 34 and Tr1 cells 35. However, so far nobody has ever tested the direct effect of G-CSF during monocyte-derived DC (moDC) differentiation.

In this report, we show that monocytes differentiated with G-CSF and IL-4 (G-DC) acquire a DC-like morphology, with up-regulation of co-stimulatory molecules, spontaneous IL-10 release, and low IL-12 production upon LPS stimulation. G-DC induce anergic but not suppressive T cells *in vitro*.

## Results

### G-CSF drives the differentiation of DC-like cells similar to post-G DC

**Figure 1:**
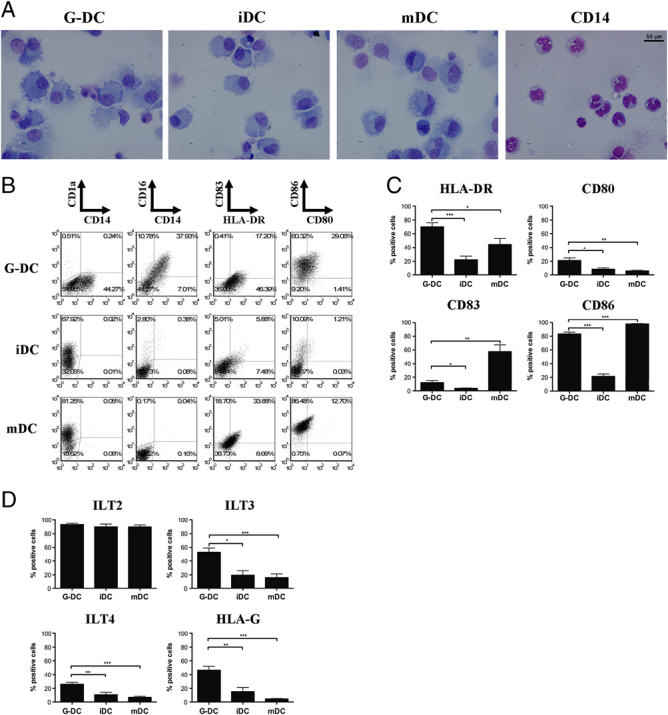
G-CSF drives the differentiation of DC-like cells similar to post-G DC. MoDC were differentiated by 7-day culture in the presence of G-CSF and IL-4 (G-DC), or GM-CSF and IL-4 (iDC), in some cases with the addition of LPS during the last 2 days of culture (mDC). (A) Cytospins were performed by centrifugation of 10^5^ cells onto slides, followed by staining with May Grünwald-Giemsa (magnification 60×). One representative donor out of four tested in two independent experiments is shown. (B–D) Expression of CD14, CD1a, CD16 (B), HLA-DR, CD80, CD83, CD86 (B and C), ILT2, ILT3, ILT4 and HLA-G (D) was evaluated by FACS on day 7. A representative donor and the mean+SEM of at least ten donors tested in five independent experiments are presented. ^*^p<0.05, ^**^p<0.01, ^***^p<0.001; Mann–Whitney test.

G-DC were differentiated from peripheral blood monocytes in the presence of G-CSF and IL-4 and compared to immature DC (iDC) differentiated with GM-CSF and IL-4 for 7 days, which in some cases were exposed to LPS during the last 2 days of culture to obtain mature DC (mDC). G-DC were differentiated in the absence of GM-CSF since it masked the effects of G-CSF in terms of phenotype and cytokine production (data not shown) 36. The yield of G-DC was 60±7.5% less than iDC (*n*=38). A small portion of G-DC cultures maintained monocyte features, while most cells became large and granular; neither of the two populations attached to the plate (data not shown). The most represented portion of G-DC displayed a DC-like morphology with eccentric nucleus, large cytoplasm and tiny protrusions, and was clearly distinct from monocytes, which were smaller and with the typical bean-shaped nucleus. G-DC were similar to iDC, although larger, with more granules and dendrites (Fig. 1A). Moreover, G-DC did not express CD115 and M-DC8 (data not shown).

G-DC expressed CD14 but not CD1a (Fig. 1B, upper panels), as already reported for post-G DC 32, HGF-conditioned DC 36 and DC-10 37,38. Interestingly, some CD14^+^ cells also co-expressed CD16 (Fig. 1B, lower panels). G-DC were HLA-DR^+^CD80^+^CD83^+^CD86^+^ even in the absence of maturation stimuli (Fig. 1C). G-DC expressed significantly higher levels of HLA-DR (70% *versus* 22%, *p*=0.0004), CD80 (21% *versus* 7%, *p*=0.0257), CD83 (on average 11% *versus* 4%, *p*=0.0404), and CD86 (on average 83% *versus* 21%, *p*<0.0001) compared to iDC (Fig. 1C). Conversely, compared to mDC, G-DC expressed significantly higher levels of HLA-DR (on average 70% *versus* 44%, *p*=0.0332) and CD80 (21% *versus* 6%, *p*=0.0020), but lower levels of CD83 (on average 11% *versus* 58%, *p*=0.0040) and CD86 (on average 83% *versus* 97%, *p*<0.0001; Fig. 1C). Interestingly, G-DC did not significantly up-regulate co-stimulatory molecules and maturation markers upon LPS stimulation (Supporting Information Fig. 1). Thus, G-DC are phenotipically similar to post-G and HGF-conditoned DC, which are able to induce Tr1 cell differentiation 32 and expansion of FOXP3^+^ Treg 36, respectively. Therefore, we investigated the expression of tolerogenic markers involved in Treg induction on G-DC. ILT2 was expressed at high levels by G-DC, iDC and mDC (Fig. 1D). Interestingly, G-DC expressed significantly higher levels of ILT3 (53% *versus* 16%, *p*=0.0009), ILT4 (26% *versus* 6%, *p*<0.0001), and HLA-G (46% *versus* 4%, *p*<0.0001) compared to mDC. Moreover, G-DC expressed higher levels of ILT3 (53% *versus* 20%, *p*=0.0206), ILT4 (26% *versus* 11%, *p*=0.0046) and HLA-G (46% *versus* 15%, *p*=0.0087) compared to iDC (Fig. 1D). PD-L1, PD-L2, ICOS-L and CD123 were not up-regulated on G-DC compared to iDC (data not shown).

### G-DC spontaneously produce IL-10 and upon activation secrete low levels of IL-12

**Figure 2:**
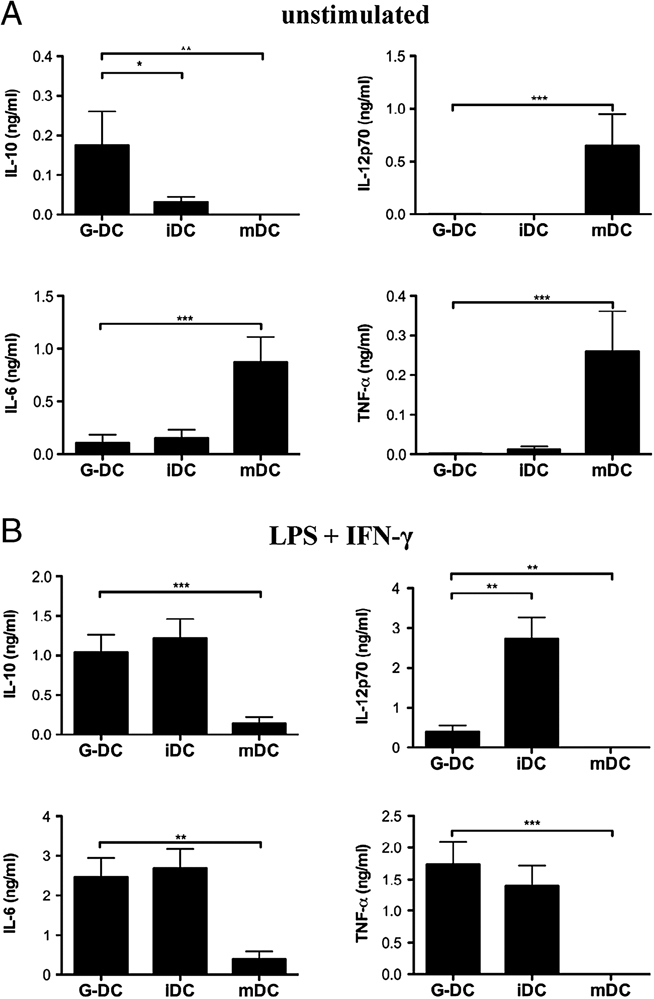
G-DC spontaneously produce IL-10 and upon activation secrete low levels of IL-12. MoDC were differentiated by 7-day culture in the presence of G-CSF and IL-4 (G-DC), or GM-CSF and IL-4 (iDC), in some cases with the addition of LPS during the last 2 days of culture (mDC). (A and B) DC were washed and seeded in the absence (A) or presence (B) of LPS and IFN-γ; supernatants were collected 48 h later to measure cytokine release. Data show mean+SEM of at least 12 donors tested in six independent experiments. ^*^p<0.05, ^**^p<0.01; Mann–Whitney test.

Similar to iDC, G-DC did not produce IL-12 or TNF-α if not stimulated, and spontaneously produced IL-6 (102±82 *versus* 152±77 pg/mL). Interestingly, G-DC spontaneously secreted higher levels of IL-10 compared to iDC (on average 175±85 *versus* 43±21 pg/mL, *p*=0.0386). Compared to mDC, unstimulated G-DC secreted significantly lower levels of IL-6 (102±82 *versus* 875±233 pg/mL, *p*=0.0008), but significantly higher levels of IL-10 (175±85 *versus* 0 pg/mL, *p*=0.0002; Fig. 2A).

Upon stimulation, G-DC released similar amounts of IL-10 (1±0.2 *versus* 1.5±0.4 ng/mL), IL-6 (2.5±0.5 *versus* 2.7±0.5 ng/mL), and TNF-α (1.7±0.4 *versus* 1.4±0.3 ng/mL), but lower levels of IL-12p70 (0.4±0.2 ng/mL *versus* 2.7±0.5 ng/mL, *p*=0.0025), compared to activated iDC. Upon stimulation, mDC did not produce any cytokine, apart from low levels of IL-6 and IL-10, a feature not due to cell death (as ruled out by trypan blue exclusion and/or annexin V/propidium iodide analyses; data not shown), but more likely to cell exhaustion (Fig. 2B). Therefore, in the steady state G-DC produce IL-10, and low levels of IL-12 upon activation.

### G-DC display hypo-stimulatory capacity and promote T-cell anergy

**Figure 3:**
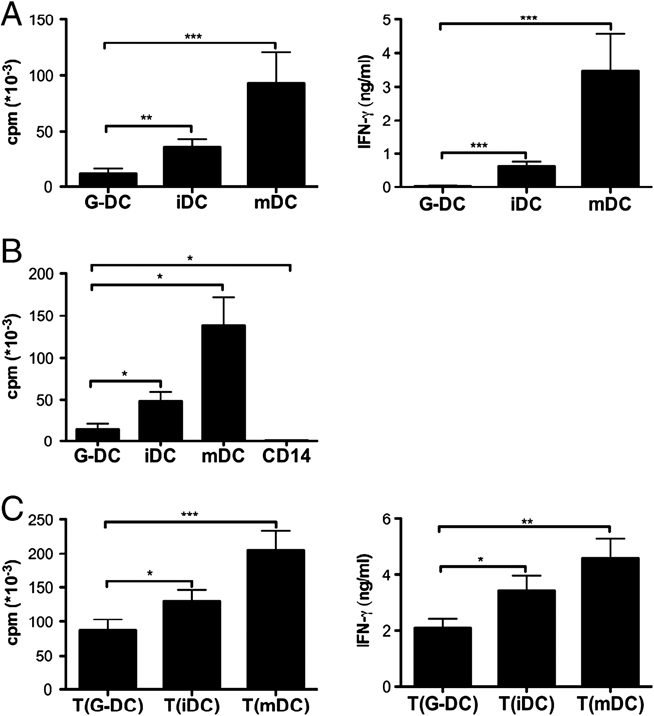
G-DC display hypo-stimulatory capacity and promote T-cell anergy. Naïve CD4^+^ T cells were primed with irradiated (6000 rad) allogeneic G-DC, iDC, or mDC at 10:1 ratio. (A) After 4 days, 50 μL of supernatant were taken to test IFN-γ release (right) and cells were pulsed for 16 h with 1 μCi/well ^3^H-thymidine (left). Data show mean+SEM of 11 donors for proliferation and 13 donors for IFN-γ production, tested in seven independent experiments. (B) Naïve CD4^+^ T cells were cultured with irradiated allogeneic DC or CD14^+^ monocytes at 1:1 ratio. After 4 days, cells were pulsed for 16 h with 1 μCi/well ^3^H-thymidine. Data show mean+SEM of three donors. (C) After 14 days of culture, T cells primed with G-DC [T(G-DC)], iDC [T(iDC)], or mDC [T(mDC)] were washed and evaluated for their proliferative response to mDC from the same allogeneic donor. After 2 days, 50 μL of supernatant were taken to test IFN-γ release (right) and cells were pulsed for 16 h with 1 μCi/well ^3^H-thymidine (left). Data show mean+SEM of 14 donors tested in eight independent experiments. ^*^p<0.05, ^**^p<0.01, ^***^p<0.001; Mann–Whitney test.

Priming of naïve CD4^+^ T cells with allogeneic G-DC at 10:1 ratio resulted in very low T-cell proliferation (on average 85%, range 46–99%, less than the proliferation induced by mDC, *p*=0.0005). As reference, iDC induced 55% (range 31–89%) less T-cell proliferation than mDC (*p*=0.0068 between G-DC and iDC; Fig. 3A, left). Similarly, naïve CD4^+^ T cells primed with allogeneic G-DC produced 98% less IFN-γ (range 83–100%) compared to T cells primed with mDC (*p*<0.001), while iDC induced 82% less IFN-γ compared to mDC (range 20–93%; *p*=0.001 between G-DC and iDC; Fig. 3A, right). Thus, G-DC have lower stimulatory capacity compared to iDC.

Notably, at 1:1 ratio, naïve T cells primed with allogeneic G-DC showed a proliferative response, although lower compared to T cells primed with either iDC or mDC, but significantly higher compared to T cells primed with CD14^+^ monocytes (*p*=0.05 for all statistic analyses shown in Fig. 3B). Thus, G-DC have the functional properties of DC, since they can prime naïve T cells.

Naïve CD4^+^ T cells primed with allogeneic G-DC [T(G-DC)], iDC [T(iDC)], or mDC [T(mDC)] were re-stimulated with mDC from the same allogeneic donor to assess secondary responses. G-DC induced anergy in T cells, which proliferated on average 57% (range 37–78%) less than T(mDC) cells (*p*=0.0005). As reference, T cells stimulated with iDC [T(iDC)] proliferated 36% less than T(mDC) cells upon re-stimulation (range 0–60%; *p*=0.0180 between T(iDC) and T(G-DC); Fig. 3C, left). When supernatants were collected from the co-cultures, IFN-γ production by T(G-DC) cell lines was on average 63% lower (range: 18–98%) compared to that by T(mDC) cell lines (*p*=0.0060). As reference, T(iDC) cells produced on average 26% less IFN-γ (range 10–75%) compared to T(mDC) cells (*p*=0.0416; Fig. 3C, right). In conclusion, G-DC display very low T-cell stimulatory capacity and induce anergy in naïve T cells. Notably, T(G-DC) cell anergy was reverted by the addition of IL-2 to the culture (data not shown).

### T cells primed with G-DC acquire a Tr1-like cytokine profile but do not suppress *in vitro*

**Figure 4:**
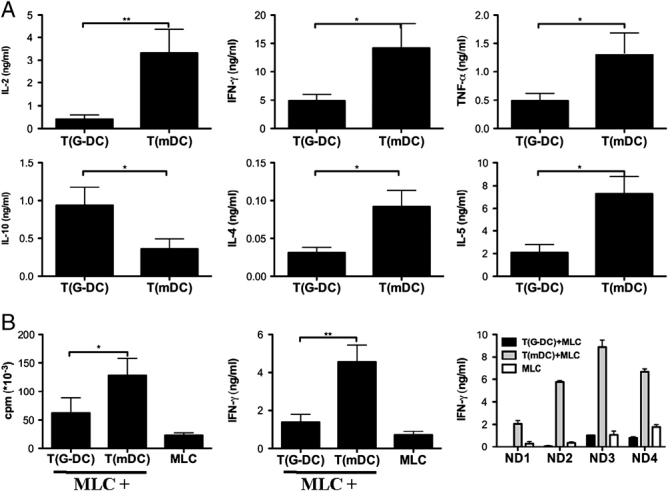
T cells primed with G-DC acquire a Tr1-like cytokine profile but not suppressive capacity. Naïve CD4^+^ T cells were cultured with irradiated (6000 rad) allogeneic G-DC [T(G-DC)] or mDC [T(mDC)] at 10:1 ratio. (A) After 14 days, T-cell lines were washed and re-stimulated with mDC from the same allogeneic donor. Culture supernatants were collected at 24 h (IL-2) and 48 h (IL-4, IL-5, IL-10, IL-17, IFN-γ and TNF-α), and cytokine levels were evaluated by Bioplex. Data show mean+SEM of eight donors tested in four independent experiments. (B) After 14 days of culture, T-cell lines were washed and evaluated for their ability to suppress the proliferation of autologous CD4^+^ T cells activated by mDC from the same allogeneic donor (MLC). After 3 days, 50 μL of supernatant were taken to test IFN-γ release (middle and right) and cells were pulsed for 16 h with 1 μCi/well ^3^H-thymidine (left). Data show mean+SEM of five donors for proliferation and eight donors for IFN-γ production, tested in three to six independent experiments (left and middle), and the production of IFN-γ by the four suppressive donors (right). ^*^p<0.05, ^**^p<0.01, ^***^ *p*<0.001; Mann–Whitney test. ND: normal donor.

Upon re-stimulation with mDC from the same allogeneic donor used for their priming, T(G-DC) cells secreted significantly higher levels of IL-10 compared to T(mDC) cells (0.94±0.24 *versus* 0.36±0.13 ng/mL, *p*=0.035; Fig. 4A). T(G-DC) cell lines secreted also substantial levels of IFN-γ, IL-2, TNF-α and IL-5, and very low levels of IL-4; however, cytokine levels remained significantly lower compared to those secreted by T(mDC) cell lines (4.9±1.1 *versus* 14.2±4.3 ng/mL of IFN-γ, *p*=0.0359; 0.4±0.2 *versus* 3.3±1 ng/mL of IL-2, *p*=0.0047; 0.5±0.1 *versus* 1.3±0.4 ng/mL of TNF-α, *p*=0.0396; 2.1±0.7 *versus* 7.2±1.5 ng/mL of IL-5, *p*=0.0190; 0.031±0.006 *versus* 0.092±0.021 ng/mL of IL-4, *p*=0.0173). IL-17 was barely detectable in all conditions tested (data not shown). Upon polyclonal stimulation, IL-10 release by T(G-DC) cells was reduced compared to allo-specific stimulation and reached the same levels of IL-10 released by polyclonally stimulated T(mDC) cells (0.54±0.17 *versus* 0.45±0.18 ng/mL; Supporting Information Fig. 2); no statistically significant differences were found in IL-2, IFN-γ, TNF-α and IL-5 production between T(G-DC) and T(mDC) cells. Therefore, T(G-DC) cells display a Tr1-like cytokine profile upon allo-specific stimulation.

We next investigated the suppressive ability of T(G-DC) cells. On average (*n*=5), proliferation of autologous CD4^+^ T cells stimulated with allogeneic mDC (MLC) was not reduced by the addition of T(G-DC) cells, although the overall proliferation was substantially lower than that observed in cultures with mDC (*p*=0.0040; Fig. 4B, left). Only T(G-DC) cells from one out of five donors were able to suppress the proliferation of the MLC (about 72% of suppression; data not shown). On average (*n*=8), addition of T(G-DC) cells to the MLC increased the overall levels of IFN-γ compared to the MLC alone. However, levels of IFN-γ in T(G-DC)+MLC co-cultures were significantly lower compared to those in T(mDC)+MLC co-cultures (*p*=0.0079; Fig. 4B, middle). Of notice, in four out of eight donors tested, individually shown in Fig. 4B, right, addition of T(G-DC) cells to the MLC suppressed IFN-γ release (on average 60% reduction, range 4–100%, of IFN-γ production in T(G-DC)+MLC co-cultures compared to the MLC alone, Fig. 4B, right), indicating that the tolerogenic potential of G-CSF varies in different donors, likely because of the genetic background of the host 39. Altogether, these results indicate that T(G-DC) cells, despite their Tr1-like cytokine profile, do not acquire full suppressive capacity typical of Tr1 cells. The percentage of CD4^+^CD25^+^FOXP3^+^ T cells was not increased in T(G-DC) cultures (data not shown), indicating that G-DC did not induce the expansion of natural Treg.

### Addition of IL-10, anti-IL-12 and anti-TNF-α Ab does not rescue the suppressive ability of T(G-DC) cells

**Figure 5:**
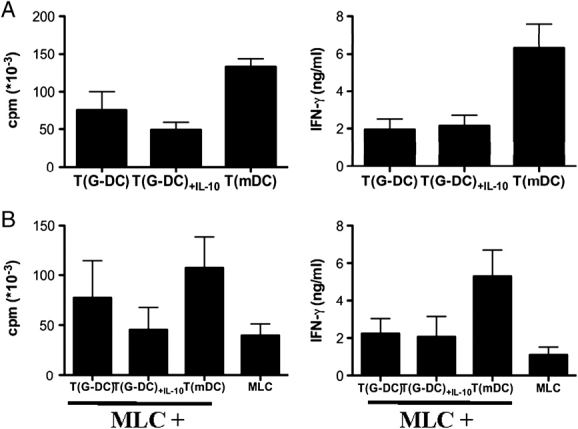
Addition of IL-10, anti-IL-12 and anti-TNF-α antibodies does not rescue the suppressive ability of T(G-DC) cells. Naïve CD4^+^ T cells were cultured with allogeneic G-DC at 10:1 ratio, in the absence [T(G-DC)] or presence [T(G-DC)_+IL-10_] of exogenous IL-10 and blocking antibodies against IL-12 and TNF-α. (A) After 14 days of culture, T-cell lines were washed and evaluated for their proliferative response to mDC from the same allogeneic donor. After 2 days, 50 μL of supernatant were taken to test IFN-γ release (right) and cells were pulsed for 16 h with 1 μCi/well ^3^H-thymidine (left). Data show mean+SEM of five donors for proliferation and seven donors for IFN-γ production, tested in three to four independent experiments. (B) After 14 days of culture, T-cell lines were washed and evaluated for their ability to suppress the proliferation of autologous CD4^+^ T cells activated by mDC from the same allogeneic donor (MLC). After 3 days, 50 μL of supernatant were taken to test IFN-γ release (right) and cells were pulsed for 16 h with 1 μCi/well ^3^H-thymidine (left). Data show mean+SEM of three donors tested in two independent experiments.

We hypothesized that the insufficient release of IL-10 by G-DC, coupled with the production of pro-inflammatory cytokines, could contribute to their failure in inducing Tr1 cells. To prove this hypothesis, we differentiated naïve T cells with G-DC in the presence [T(G-DC)_+IL-10_] or absence [T(G-DC)] of exogenous IL-10 (10 ng/mL) and neutralizing antibodies against IL-12 and TNF-α. When re-stimulated with the same allo-antigen used for their priming, T(G-DC) cells proliferated 52% less than T(mDC) cells, while T(G-DC)_+IL-10_ cells proliferated 62% less than T(mDC) cells (Fig. 5A, left). No differences were found between T(G-DC) cells and T(G-DC)_+IL-10_ cells in terms of IFN-γ release (71% *versus* 67% less IFN-γ than T(mDC) cells, respectively; Fig. 5A, right).

T(G-DC)_+IL-10_ cells did not show any suppressive ability on autologous T cells when added to the MLC (Fig. 5B, left). Similar data were obtained for IFN-γ production (Fig. 5B, right). Of notice, T(G-DC) cells from one out of the three donors tested were able to suppress the proliferation of autologous T cells independently of the presence of exogenous IL-10 and neutralizing antibodies (data not shown), in line with the variability observed in the previous suppressive assay. Therefore, G-DC were unable to induce T cells with suppressive capacity even in these culture conditions.

## Discussion

We demonstrate that G-CSF, in combination with IL-4, promotes the generation of a population of moDC, here called G-DC, exhibiting a peculiar phenotype. G-DC express CD14 and CD16 but not CD1a, are HLA-DR^+^CD80^+^CD83^+^CD86^+^, and express tolerogenic markers (ILT4 and HLA-G) involved in Tr1 cell induction 37,38. G-DC display hypo-stimulatory capacity and induce anergic but not suppressive T cells *in vitro*.

We show that G-CSF not only directly induces moDC differentiation, but also modulates their phenotype and functions *in vitro*. Rutella *et al.* demonstrated that monocytes from G-CSF-treated healthy donors, exposed to autologous (post-G) serum in the absence of exogenous cytokines, differentiate into DC-like cells (post-G DC) with tolerogenic features 32. G-DC share some characteristics with post-G DC 32, HGF-conditioned DC-like cells 36, DC-10 37,38 and DC conditioned with serum from cancer patients 40. These populations maintain the expression of CD14 without up-regulating CD1a, express high levels of HLA-DR and co-stimulatory molecules, produce low levels of IL-12 upon activation, and are poor stimulators.

G-DC, as well as post-G DC and DC-10, induce a Tr1-like cytokine profile and anergy in naïve T cells. However, post-G DC 32 and DC-10 37,38 induce Tr1 cells, whereas G-DC do not induce suppressive T cells. On the other hand, HGF-conditioned DC expand FOXP3^+^ Treg 36. The phenotype of post-G DC can be reproduced by culturing monocytes from G-CSF-treated healthy donors in the presence of exogenous IL-10 and IFN-α 32, while DC-10 generation requires IL-10 in combination with GM-CSF and IL-4; by contrast, G-DC are differentiated in the absence of IL-10. We can speculate that the presence of exogenous IL-10 in the culture is essential for the *in vitro* generation of tolerogenic DC able to drive Tr1 cell differentiation.

The failure of G-DC to induce suppressive T cells does not depend on defective IL-10 production, or on the release of IL-12 and TNF-α by activated G-DC; indeed, the addition of IL-10, together with blocking antibodies against IL-12 and TNF-α, during T-cell priming with G-DC does not result in the induction of suppressive Tr1 cells. Thus, other factors might contribute to the differential tolerogenic ability of post-G DC and G-DC. ILT2, ILT3 and ILT4 are well-characterized immune inhibitory receptors predominantly expressed on myeloid and plasmacytoid DC 41. IL-10 and IFN-α treatment induces ILT3 and ILT4 expression on moDC, which acquire the ability to induce Treg 42; however, another report showed that the expression of ILT3 on DC is dispensable for the induction of Treg 43. ILT3 is up-regulated on post-G DC, but its expression does not correlate with their ability to inhibit T-cell proliferation (Rutella S., personal communication). Accordingly, G-DC are not able to induce suppressive Tr1 cells despite the expression of ILT3. We recently demonstrated that the IL-10-driven up-regulation of ILT4 and HLA-G on DC-10, and HLA-G on T cells, is necessary for Tr1 cell induction 37,38. Notably, ILT4 and HLA-G were expressed at low levels by G-DC. We can speculate that the low levels of expression of ILT4 and HLA-G together with low IL-10 production by G-DC contributes to their inability to induce suppressive Tr1 cells.

It has been demonstrated that direct exposure of T cells to G-CSF *in vitro* does not induce a regulatory phenotype 19,24,25. Conversely, *in vivo* treatment with G-CSF in mouse models of stem cell transplantation 26,29,44,45 and autoimmune diseases 46–48 resulted in induction of peripheral tolerance through IL-10-producing T cells 10. Similarly, human T cells exposed to G-CSF *in vivo* acquire Tr1-like phenotype and functions 24. Therefore, indirect mechanisms triggered by G-CSF are responsible for the induction of peripheral tolerance through Treg *in vivo*. Notably, IFN-α and IL-10 are increased in the serum of G-CSF-treated healthy volunteers 32 and can be implicated in Tr1 cell induction both directly 49,50 and indirectly, by contributing to the differentiation of monocytes into tolerogenic post-G DC 32. In addition, the direct effect of G-CSF on DC could be active also *in vivo*, particularly at early time points after G-CSF administration, when the G-CSF serum levels reach the concentration used in our study 51. It is also possible that G-DC, in the presence of persistent high levels of IL-10 *in vivo*, further differentiate into DC-10, potent Tr1 cell inducers 37.

Injection of G-CSF in patients affected by Crohn's disease increases IL-10-secreting T cells in peripheral blood and enhances accumulation of plasmacytoid DC in the lamina propria, resulting in clinical benefit 52. G-CSF enhances stem cell engraftment after allogeneic HSC transplantation and prevents prolonged neutropenia, but has a neutral effect on acute GVH reaction 53 while increasing chronic GVH reaction, a phenomenon recently associated with increased Th17 differentiation 54. These apparently contrasting effects could be ascribed to the fact that G-CSF *in vivo* acts on different cell types, which are not yet reconstituted at the time of G-CSF treatment (lympho-myeloid cells), or which can be dysfunctional (stromal cells) in allogeneic HSC tranplantation but not in other settings.

In conclusion, we show for the first time that G-CSF *per se* modulates monocyte differentiation toward tolerogenic DC and directly modulates DC function. Our findings broaden the knowledge of the tolerogenic properties of G-CSF, adding a potential new mechanism of direct APC modulation, which could contribute to the induction of Treg *in vivo*.

## Materials and methods

### Cell preparation

Human peripheral blood was obtained from healthy donors in accordance with local committee approval and informed consent from all participating subjects was obtained. PBMC were separated by density gradient centrifugation over Lymphoprep (Nycomed Amersham).

### DC differentiation

CD14^+^ monocytes were isolated as the adherent fraction of PBMC following 1 h incubation in RPMI 1640 (Biowhittaker) supplemented with 10% FBS (Biowhittaker), 100 U/mL penicillin/streptomycin (Bristol-Myers Squibb), and 50 μM 2-mercaptoethanol (BioRad) (DC medium) at 37°C. Following extensive washing, adherent monocytes were cultured for 7 days in: 50 ng/mL rhG-CSF (Myelostim, Sanofi-Aventis) and 10 ng/mL rhIL-4 (R&D Systems) (G-DC); 100 ng/mL rhGM-CSF (R&D Systems) and 10 ng/mL rhIL-4 (iDC); 100 ng/mL rhGM-CSF and 10 ng/mL rhIL-4, with the addition of 1 μg/mL LPS (Sigma Aldrich) during the last 2 days of culture (mDC). On day 7, 10^5^ differentiated DC were cytospinned 5′ at 800 rpm. Slides were then stained with May Grünwald-Giemsa solutions (10′ RT). DC were tested for expression of CD1a, CD14, HLA-DR, CD11c, CD80, CD83, CD86, CD123, ILT2 (BD Biosciences), ILT3 (Coulter Immunotech), ILT4 (Beckman Coulter) and HLA-G (Exbion). ICOS-L, PD-L1 and PD-L2 were from eBioscience.

### T-cell purification and differentiation

CD4^+^ T cells were purified from PBMC by negative selection using the CD4^+^ T-cell Isolation kit (Miltenyi Biotec), according to the manufacturer's instructions. A portion of the recovered CD4^+^ T cells was cryopreserved for later use, and the remainders were depleted of CD45RO^+^ cells using anti-CD45RO-coupled magnetic beads and LD negative selection columns (Miltenyi Biotec). In the purified cells, the proportion of CD4^+^CD45RO^−^CD45RA^+^ was consistently greater than 95%. 10^5^ DC were cultured with 10^6^ allogeneic CD4^+^CD45RO^−^ T cells in 1 mL of X-VIVO 15 (Biowhittaker), supplemented with 5% pooled AB human serum (Biowhittaker), and 100 U/mL penicillin/streptomycin (Bristol-Myers Squibb). After 7 days, rhIL-2 (20 U/mL; Chiron) was added, and cells were expanded for additional 7 days. Fourteen days after initiation of the culture, T cells were collected, washed and analyzed for their functions. T cells stimulated with G-DC are referred to as T(G-DC), with iDC as T(iDC), and with mDC as T(mDC) cells. Compared to cultures stimulated with mDC, T(G-DC) cell cultures typically resulted in three- to fourfold reduction in T-cell expansion. In some experiments, T cells were cultured with G-DC in the presence or absence of IL-10 (10 ng/mL), and/or 10 μg/mL anti-TNF-α and anti-IL-12 blocking antibodies (R&D systems), and were referred to as T(G-DC)_+IL-10_ cells.

### T-cell proliferation and suppression

To determine DC stimulatory capacity, 5×10^4^ naïve T cells were stimulated with irradiated (6000 rad) allogeneic DC (10:1, T:DC; in some cases 1:1) in a final volume of 200 μL of medium in 96-well round-bottom plates. To analyze the proliferative capacity of T(G-DC), T(DC-10), or T(mDC) cell lines in response to the same allo-antigen used for their priming, T cells were stimulated with irradiated (6000 rad) allogeneic mDC (10:1) in a final volume of 200 μL of medium in 96-well round-bottom plates. In some experiments, 100 U/mL rhIL-2 were added. To test the suppressive capacity of T-cell lines, autologous CD4^+^ T cells were thawed and 5×10^4^ of the cells were stimulated with irradiated allogeneic mDC (10:1) in the absence or presence of T(G-DC), T(DC-10) or T(mDC) cells (1:1, T:T) in a final volume of 200 μL of medium in 96-well round-bottom plates. All cultures were performed in triplicate. After the indicated time, cells were pulsed for 16 h with 1 μCi/well ^3^H-thymidine.

### Cytokine determination

To measure IL-6, IL-10, IL-12p70 and TNF-α produced by DC, 5×10^4^ cells were left unstimulated or activated with 50 ng/mL rhIFN-γ (R&D Systems) and 200 ng/mL LPS (Sigma) in a final volume of 200 μL in 96-well round-bottom plates. Supernatants were collected after 48 h. Levels of IL-6, IL-10, IL-12p70 and TNF-α were determined by capture ELISA according to the manufacturer's instructions (BD Biosciences). The detection limits were as follows: IFN-γ: 60 pg/mL; IL-6: 20 pg/mL; IL-10: 20 pg/mL; IL-12: 20 pg/mL; TNF-α: 30 pg/mL. To measure IL-2, IL-4, IL-5, IL-10, IL-17, IFN-γ and TNF-α produced by T-cell lines, 5×10^4^ cells were stimulated with allogeneic mDC (10:1) or antiCD3/CD28 mAb, in a final volume of 200 μL in 96-well round-bottom plates. Supernatants were harvested after 24 h (IL-2) and 48 h (IL-4, IL-5, IL-10, IL-17, IFN-γ, and TNF-α). Cytokine levels were determined by Bioplex according to the manufacturer's instructions (Bio-Rad). The detection limits were as follows: IL-2: 4 pg/mL; IL-4: 1 pg/mL; IL-5: 6 pg/mL; IL-10: 5 pg/mL; IFN-γ: 5 pg/mL; TNF-α: 15 pg/mL.

### Statistical analysis

All statistical analyses for significant differences were performed with the non-parametric Mann–Whitney test. *P*-Values ≤0.05 were considered significant.

## Abbreviations

iDC: immature DC
ILT: Ig-like transcript
mDC: mature DC
moDC: monocyte-derived DC
post-G DC: monocytes from G-CSF-mobilized healthy donors cultured with autologous (post-G) serum
Tr1: type 1 Treg
