# Supplementary material for: Granulocyte-colony stimulating factor drives the in vitro differentiation of human dendritic cells that induce anergy in naïve T cells
Source: Eur J Immunol. 2010 Sep 21;40(11):3097–106. doi: 10.1002/eji.201040659 (PMC2997328; doi:10.1002/eji.201040659)
Supplement: Supplementary file 1 [file eji0040-3097-SD1.pdf]

European Journal of Immunology

Supporting Information

for

DOI 10.1002/eji.201040659

Granulocyte-colony stimulating factor drives the *in vitro* differentiation of human dendritic cells that induce anergy in naïve T cells

Maura Rossetti, Silvia Gregori and Maria Grazia Roncarolo

© 2010 WILEY-VCH Verlag GmbH & Co. KGaA, Weinheim

Figure 1S

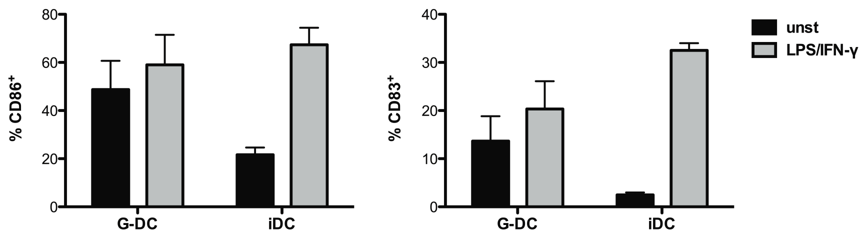

Figure 1S. G-DC do not up-regulate co-stimulatory molecules upon LPS stimulation. MoDC were differentiated by 7-day culture in the presence of G-CSF and IL-4 (G-DC), or GM-CSF and IL-4 (iDC). A-B. DC were washed and seeded in the absence (A) or presence (B) of LPS and IFN-γ; cells were stained 48 hours later for the expression of CD86 and CD83. The average +SEM of 4 donors tested in 2 independent experiments is shown.

Figure 2S

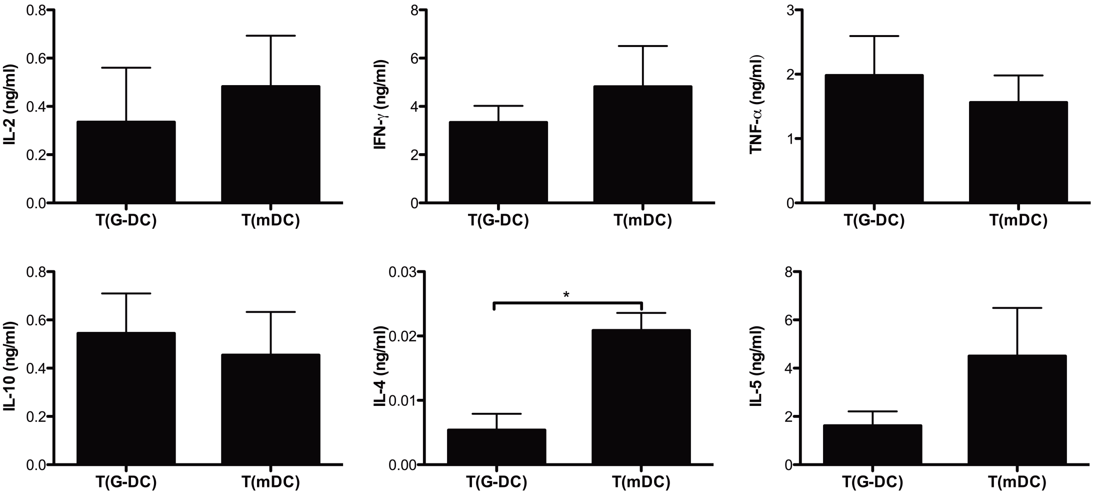

Figure 2S. T cells generated with G-DC have a similar cytokine profile compared to T cells induced by mDC upon polyclonal stimulation. Naïve $CD4^+$ T cells were cultured with allogeneic G-DC [T(G-DC)] or mDC [T(mDC)] at 10:1 ratio. After 14 days, T cell lines were washed and re-stimulated with coated anti-CD3 and soluble anti-CD28 monoclonal antibodies to test their cytokine release. Culture supernatants were collected at 24 (IL-2) and 48 hours (IL-4, IL-5, IL-10, IL-17, IFN-γ and TNF-α). The average with SEM of 10 donors tested in 5 independent experiments is shown. Statistical significance (Mann-Whitney test) of the difference between T(G-DC) cells and T(mDC) cells is reported. * when $p<0.05$.
